# Transcriptome analysis of ageing in uninjured human Achilles tendon

**DOI:** 10.1186/s13075-015-0544-2

**Published:** 2015-02-18

**Authors:** Mandy Jayne Peffers, Yongxiang Fang, Kathleen Cheung, Tim Koh Jia Wei, Peter David Clegg, Helen Lucy Birch

**Affiliations:** Comparative Musculoskeletal Biology, Institute of Ageing and Chronic Disease, University of Liverpool, Leahurst, Chester High Road, Neston, Wirral CH64 7TE UK; Centre for Genomic Research, Institute of Integrative Biology, University of Liverpool, Biosciences Building, Crown Street, Liverpool, L69 7ZB UK; Musculoskeletal Research Group, Institute of Cellular Medicine, Newcastle University, Newcastle upon Tyne, NE2 4HH UK; School of Life Sciences and Chemical Technology, Ngee Ann Polytechnic, 535 Clementi Road, Singapore, 599489 Singapore; Institute of Orthopaedics and Musculoskeletal Science, University College London, Stanmore Campus, Royal National Orthopaedic Hospital, Brockley Hill, Stanmore, HA7 4LP UK

## Abstract

**Introduction:**

The risk of tendon injury and disease increases significantly with increasing age. The aim of the study was to characterise transcriptional changes in human Achilles tendon during the ageing process in order to identify molecular signatures that might contribute to age-related degeneration.

**Methods:**

RNA for gene expression analysis using RNA-Seq and quantitative real-time polymerase chain reaction analysis was isolated from young and old macroscopically normal human Achilles tendon. RNA sequence libraries were prepared following ribosomal RNA depletion, and sequencing was undertaken by using the Illumina HiSeq 2000 platform. Expression levels among genes were compared by using fragments per kilobase of exon per million fragments mapped. Differentially expressed genes were defined by using Benjamini-Hochberg false discovery rate approach (*P* <0.05, expression ratios 1.4 log_2_ fold change). Alternative splicing of exon variants were also examined by using Cufflinks. The functional significance of genes that showed differential expression between young and old tendon was determined by using ingenuity pathway analysis.

**Results:**

In total, the expression of 325 transcribed elements, including protein-coding transcripts and non-coding transcripts (small non-coding RNAs, pseudogenes, long non-coding RNAs and a single microRNA), was significantly different in old compared with young tendon (±1.4 log_2_ fold change, *P* <0.05). Of these, 191 were at higher levels in older tendon and 134 were at lower levels in older tendon. The top networks for genes differentially expressed with tendon age were from cellular function, cellular growth, and cellular cycling pathways. Notable differential transcriptome changes were also observed in alternative splicing patterns. Several of the top gene ontology terms identified in downregulated isoforms in old tendon related to collagen and post-translational modification of collagen.

**Conclusions:**

This study demonstrates dynamic alterations in RNA with age at numerous genomic levels, indicating changes in the regulation of transcriptional networks. The results suggest that ageing is not primarily associated with loss of ability to synthesise matrix proteins and matrix-degrading enzymes. In addition, we have identified non-coding RNA genes and differentially expressed transcript isoforms of known matrix components with ageing which require further investigation.

**Electronic supplementary material:**

The online version of this article (doi:10.1186/s13075-015-0544-2) contains supplementary material, which is available to authorized users.

## Introduction

The increasing number of people reaching old age provides huge challenges to society, as whereas life span increases, life quality faced by many individuals in old age is poor [1]. Whereas muscle, bone, and joint age-related disease is well recognised, the fibrous connecting tendon tissue has received little attention, despite representing a very common site of pain and dysfunction. Epidemiological studies have revealed a clear link between age and increasing incidence of tendon injury [2,3], suggesting that the mechanical integrity of tendon declines during the ageing process.

Although it is generally accepted that a degenerative process precedes gross tendon injury, the aetiology of this process remains elusive and the definition of degeneration is poorly defined. Histological examination of painful Achilles tendon [4], dysfunctional posterior tibialis [5], and supraspinatus tendon collected from cadavers [6] has revealed pathological changes, including signs of collagen fibre disruption, increased staining for glycosaminoglycan, hypercellularity, and cell shape change to a more chondroid appearance. Similar changes have been observed in macroscopically abnormal equine flexor tendon [7], another common site of age-associated tendon injury. Histological abnormalities are more often observed in older individuals [6], although the relationship with ageing and the apparent change in cell function is not clear.

Ageing is generally associated with a decline in protein synthesis [8] and a loss of cell functionality [9]. It has been suggested that early degenerative changes in tendon result from an accumulation of micro-damage within the extracellular matrix (ECM) due to an imbalance between anabolic and catabolic pathways [10]. Recent work on equine flexor tendon identified an accumulation of partially degraded collagen within the ECM of old tendons, and it was hypothesised that an inability to remove partially degraded collagen may account for reduced mechanical competency [11]. Another study found that flexor tendon explants from older horses were more susceptible to fatigue damage following cyclical loading *in vitro* than explants from young horses and that this was a cell-mediated process involving the matrix metallo-proteinases (MMPs) [12].

Cell ageing has been associated with a decreased ability to modulate inflammation resulting in a chronic low-level inflammation termed ‘inflamm-aging’ [13]. Recent work by Dakin and colleagues [14] measured prostaglandin E2 in injured equine flexor tendons and found that levels increased with increasing horse age but that levels of formyl peptide receptor 2/ALX, a receptor responsible for suppressing the inflammatory response, were significantly reduced. These findings intimate that aged individuals exhibit a reduced capacity to resolve inflammation and that ageing may contribute to deregulated tendon repair through these pathways.

Quantitative analysis of gene expression changes with age may help the understanding of ageing mechanisms and their interactions with age-related diseases such as tendinopathies [15]. Although microarray technology has been employed to investigate gene expression changes following tendon injury [16], in tendinopathic tissue [17], in response to cyclical strain [18] or a single loading event [19], and effect of loading on tendon healing [20], no comprehensive analysis of alterations in gene expression with age has been undertaken in tendon.

RNA-Seq can capture the whole transcriptome, including coding RNAs, isoforms produced by alternative splicing, long non-coding RNAs (lncRNAs) (the importance of which is becoming apparent in disease [21] and ageing [22-24]), and short non-coding RNAs. We have previously used RNA-Seq successfully on equine cartilage tissue and identified an over-representation of genes with reduced expression relating to ECM, degradative proteases, matrix synthetic enzymes, cytokines, and growth factors in ageing cartilage [24].

In this study, we used RNA-Seq to comprehensively identify the human Achilles tendon transcriptome for the first time and then examine changes that occur with ageing. We hypothesised that ageing results in reduced expression of ECM-related proteins and matrix-degrading enzymes. In addition, we sought to identify previously unrecognised slice variants and non-coding RNAs associated with tendon ageing in a ‘bottom-up’ inductive approach.

## Methods

### Sample collection and preparation

All human Achilles tendons used in this study—RNA-Seq and quantitative real-time polymerase chain reaction (qRT-PCR)—were harvested from limbs amputated during surgical procedures to treat sarcomas at the Royal National Orthopaedic Hospital, Stanmore. Tissue collection was carried out through the Stanmore Musculoskeletal BioBank, which has ethical approval from the Cambridgeshire 1 Research Ethics Committee (REC reference 09/H0304/78) to collect tissue for research into musculoskeletal conditions. All patients gave consent for their tissue to be used for musculoskeleton-related research. Local research-and-development approval for this project was given by the UCL/UCLH/RF Joint Research Office (reference number 11/0464). For RNA-Seq, tendons were collected from donors who were 69.4 ± 7.3 years old (old group, n = 5, 3 female, 2 male) and donors who were 19 ± 5.8 years old (young group, n = 4, 4 male). Tendon tissue was collected within 24 hours of limb removal, except for one sample in which tissue was collected within 48 hours. The Achilles tendon was dissected free from the limb. Only tendons with a normal macroscopic appearance were used for this study. A section of tissue approximately 1 cm in length was taken from the mid region of the tendon between the musculotendinous junction and the insertion site. Outer tissue (paratenon) was removed and the remaining tendon tissue placed into RNAlater (Ambion, Warrington, UK) in accordance with the instructions of the manufacturer.

### RNA extraction

Tendon was pulverising into a powder with a dismembranator (Mikro-S; Sartorius, Melsungen, Germany) following freezing in liquid nitrogen. Immediately, 20 volumes of Tri Reagent (Ambion) was added to the powdered tendon tissue and the RNA extracted and purified as described by Peffers *et al*. [25] (2013). RNA was quantified by using a Nanodrop ND-100 spectrophotometer (Labtech, Uckfield, East Sussex, UK) and assessed for purity by UV absorbance measurements at 260 and 280 nm.

### RNA-Seq analysis: cDNA library preparation and sequencing

Total RNA was analysed by the Centre for Genomic Research, University of Liverpool, for RNA-Seq library preparation and sequencing by using the Illumina HiSeq 2000 platform. Total RNA integrity was confirmed by using an Agilent 2100 Bioanalyzer (Agilent Technologies, Santa Clara, CA, USA). Ribosomal RNA (rRNA) was depleted from 9 total RNA samples by using the Ribo-Zero™ rRNA Removal Kit (Human/Mouse/Rat; Epicentre, Madison, WI, USA) in accordance with the instructions of the manufacturer. cDNA libraries were prepared with the ScriptSeq v2 RNA-Seq library preparation kit (Epicentre) by using 50 ng rRNA depleted RNA as starting material in accordance with manufacturer protocols as previously described [24]. The final pooled library was diluted to 8 pmol before hybridisation. The dilute library (120 μL) was hybridised on one lane of the HiSeq 2000 at 2 × 100-base pair (bp) paired-end sequencing with v3 chemistry.

### Data processing

The sequence libraries for each sample were processed by using CASAVA version 1.8.2 to produce 100-bp paired-end sequence data in fastq format. The fastq files were processed by using Cutadapt version 1.2.1 [26] with option ‘–O 3’ to trim adapter from any read if it matched the adapter sequence for 3 bp or more at the 3′ end. In addition, a quality trimming was performed by using Sickle version 1.200.

The trimmed R1-R2 read pairs, for each sample, were aligned to reference sequence [27] by using TopHat2 version 2.0.10 [28] with default settings, except for the option –g 1. Read counts were obtained from the mapping results by using HTSeq-count and genome annotation [29].

The differential gene expression analysis was performed on R platform by using the edgeR package [30] and focused on the contrast of old and young donors. The count data were normalised across libraries by using trimmed mean M (TMM) values of the default methods edgeR. The tagwise dispersions were estimated and then used for logFC (log_2_ fold change) estimating and testing. Differentially expressed genes (DEGs) were extracted by applying the threshold false discovery rate (FDR) of less than 0.05 to adjusted *P* values, which were generated by using Benjamini and Hochberg approach [31]. In addition, FPKM (fragments per kilobase of exon per million fragments mapped) values were converted from count values for comparing expression levels among genes. All sequence data produced in this study have been submitted to National Center for Biotechnology Information Gene Expression Omnibus (NCBI GEO) under Array Express accession number E-MTAB-2449.

### Analysis of splice variants

Trimmed paired reads were aligned to a reference human transcriptome (Ensembl iGenomes build GRCh37) by using Bowtie2 [32]. The alignments (BAM files) were converted into sorted SAM files by using SAMtools [33]. Parameters for TopHat were estimated by using a Picard tool (CollectInsertSizeMetrics.jar) [34]. Reads were aligned to the reference genome (Ensembl build GRCh37) by using TopHat [35], specifying mate inner distance (mean inner distance between mate pairs) and standard deviation for each sample. Mapped reads were then assembled into complete transcripts by using the splice junction mapping tool Cufflinks [36] with option –G, which uses the Ensembl reference gene track to improve mapping. Cuffmerge was used to merge the assembled transcripts into a consensus gene track from the all of the mapped samples. Cuffdiff was used to identify DEGs and differentially expressed transcripts between young and old tendon. Genes and transcripts were identified as being significantly differentially expressed with q values of less than 0.05, calculated by the Benjamini and Hochberg FDR correction [31].

Downstream analysis and visualisation of results, including quality control of the samples, was undertaken by using the cummeRbund package in R. Graphs were generated by using cummeRbund and the ggplot2 package [37].

### Functional analysis

To systematically determine networks, functional analyses, and canonical pathways that the DEGs might involve, we performed the pathway/network enrichment analysis using the ingenuity pathway analysis (IPA) tool from the Ingenuity Systems [38] by using a list of DEGs with values-adjusted *P* value of less than 0.05 and ±1.4 log_2_ fold regulation. Gene symbols were used as identifiers and the Ingenuity Knowledge Base gene was used as a reference for a pathway analysis. For network generation, a data set containing gene identifiers and corresponding expression values was uploaded. Default settings were used to identify molecules whose expression was significantly differentially regulated. These molecules were overlaid onto a global molecular network contained in the Ingenuity Knowledge Base. Networks of ‘network-eligible molecules’ were then algorithmically generated based on their connectivity. The functional analysis identified the biological functions and diseases that were most significant to the data set. Right‐tailed Fisher’s exact test was used to calculate *P* values. Canonical pathways analysis identified the pathways from the IPA library that were most significant to the data set.

For isoform analysis, the Database for Annotation, Visualization and Integrated Discovery (DAVID) (DAVID bioinformatics resources 6.7) was used [39]. The web-based functional annotation tool enabled functional clustering of gene. The functional clustering tool was used for functional enrichment for DEG isoforms with values-adjusted *P* value of less than 0.05 and ±1.4 log_2_ fold regulation.

### Real-time polymerase chain reaction

Samples of RNA from both the same pools used for the RNA-Seq analysis and an additional independent cohort harvested in the same manner (n = 4 young; 16.7 ± 2.8 years old and n = 4 old; 73.2 ± 6.5 years old) were used for qRT-PCR. To validate results from differentially expressed isoforms, the independent cohort was used. Moloney murine leukaemia virus (M-MLV) reverse transcriptase and random hexamer oligonucleotides (both from Promega, Southampton, UK) were used to synthesize cDNA from 1 μg RNA in a 25 μL reaction. PCR was performed on 1 μL 10× diluted cDNA by employing a final concentration of 300 nM of each primer in 20 μL reaction volumes on an ABI 7700 Sequence Detector using PrimerDesign 2X PrecisionTM SYBR Green Mastermix (Primer Design, Southampton, UK). qRT-PCR was undertaken by using gene-specific primers (for protein-coding genes these were exon-spanning). Primers used had been validated in previous publications [40,41] and supplied by Eurogentec (Seraing, Belgium) or were designed and validated commercially (Primer Design). Steady-state transcript abundance of potential endogenous control genes was measured in the RNA-Seq data. Assays for four genes—glucose-6-phosphate isomerise (GPI), beta-actin (ACTB), ribosomal protein 13 (RSP13), and ribosomal protein 16 (RPS16)—were selected as potential reference genes as their expression was unaltered. Stability of this panel of genes was assessed by applying a gene stability algorithm [42]. RSP16 was selected as the most stable endogenous control gene. Relative expression levels were normalised to RPS16 and calculated by using the 2^−∆Ct^ method [43]. Primers pairs used in this study are listed (Table 1). qRT-PCR analysis data was log_10_-transformed to ensure normal distribution and then analysed by using Student’s *t* test.

**Table 1:** **Gene primer sequences used in RNA-Seq validation**

**Name**	**Gene type**	**Ensemble gene ID/transcript ID**	**Primer sequence**
*RPS16*	Protein-coding	ENSG00000105193	F: GAAATCCTACCGATAAGCCCA
			R: TTTCTTGAAACTTTAAAATCCCTCAA
*MMP3*	Protein-coding	ENSG00000149968	F: ATTCCATGGAGCCAGGCTTTC
			R: CATTTGGGTCAAACTCCAACTGTG
*COL1A1* ^*a*^	Protein-coding	ENSG00000108821	F: GGAGGAGAGTCAGGAAGG
			R: GCAACACAGTTACACAAGG
*ACAN* ^*b*^	Protein-coding	ENSG00000108821	F: TCGAGGACAGCGAGGCC
			R: TCGAGGGTGTAGCGTGTAGAGA
*POU3F4*	Protein-coding	ENSG00000196767	F: GTTCGCTCGCTCTCTCGTA
			R: GAAGGGAAGGGAAGGGGAAA
*MYF5*	Protein-coding	ENSG00000111049	F: TGAACTAATTTTCTGGTCTATATGAC
			R: TGTACATGTTGTCTTGGTTTGGG
*IGF1*	Protein-coding	ENSG00000017427	F: CACTATGGACAGATGTAAAAGAAACTA
			R: ACACACTGGGGACAAGAAATAAA
*MMP16*	Protein-coding	ENSG00000156103	F: ACCCGTGTAACCCTTTGAGA
			R: AACCTGAACTTCTTGAACTTGTG
*TGFB3*	Protein-coding	ENSG00000119699	F: GTAAAGAAAGTGTGGGTTTGGTTA
			R: AACATCTCAACTTACCATCCCTTT
*EGF*	Protein-coding	ENSG00000138798	F: ACAGGAGGCTTCGGAGTT
			R: AATCAGGCAATTTACTTACAATCTT
*COL3A1-001*	Protein-coding	ENST00000304636	F: CAGGTCCCAGCGGTTCT
			R: CCTTTTGGTCCAGACACTCC
*COL3A1-201*	Protein-coding	ENST00000317840	F: GGTAGCCCTGGTGAGAGAG
			R: TGCCAGGAGGTCCAAAGAG
*FGF10-001*	Protein-coding	ENST00000264664	F: TGCCGTCAAAGCCATTAACA
			R: CATTTTCCTCTATCCTCTCCTTCA
*CRTAC1-001*	Protein-coding	ENST00000370597	F:ATCTTCTTCAACAACATTGCCTAC
			R: GGGTCTCCGTGCTCTCTAC
*IGF1-001*	Protein-coding	ENST00000337514	F: CAGCAGTCTTCCAACCCAAT
			R: AAGAGATGCGAGGAGGACAT
*XIST*	Lnc	ENSG00000229807	F: TCCCAGAGAATGCCTAATACTTT
			R: GTAGAAGAGATACGGAGTAGGAAT
*LINC00957*	Lnc	ENSG00000235314	F: GAGAGTAAGCAGACCTGGGT
			R: ACCTTGTCCGAGTTCCATCT
*RP11.308 N19.1*	Lnc	ENSG00000234323	F: GCCTCTTTCATCACTGCCGA
			R: TAGCAGCAGTTGGGGTGTTT

^a^[40] and ^b^[41] denote primer pairs published previously. F, forward; Lnc, long non-coding; R, reverse.

### Statistical analysis

The analyses were undertaken by using edgeR [30], S-Plus (version 7.0; Tibco Software Inc., Palo Alto, CA, USA), SPSS (version 20; IBM, Portsmouth, Hampshire, UK), and Excel (2007; Microsoft, Redmond, WA, USA) software.

## Results

### Overview of RNA-Seq data

An average of 32.1 million pairs of 100-bp paired-end reads per sample were generated that aligned to the reference sequence of the human genome. Using pooled R1 and R2 files for all samples in Trimmed data gave 95.1% of called bases with of Phred score of more than 30 [44]. (See Table 2 for summary of mapping results.) Of the 63,152 human genes, between 40.5% and 47.4% had at least one read aligned; 20,322 of the genes had no reads aligned from any of the nine samples. This is similar to the output of other RNA-Seq sequencing studies [24,45].

**Table 2:** **Summary of sequence alignment to the human genome**

**Sample**	**Reads to align** ^**a**^	**Reads aligned to genome**	**Percentage** ^**b**^	**Reads properly paired**	**Percentage** ^**c**^	**Reads with mate unmapped**	**Percentage** ^**d**^	**Percentage of ‘expressed’ genes** ^**e**^
Young 1	61,516,106	55,995,223	91.03	51,821,068	92.55	4,174,155	7.45	46.65
Young 2	74,068,430	67,565,304	91.22	62,477,226	92.47	5,088,078	7.53	45.98
Young 3	65,954,168	60,771,423	92.14	56,659,046	93.23	4,112,377	6.77	47.19
Young 4	59,947,540	53,381,917	89.05	48,909,710	91.62	4,472,207	8.38	40.58
Young 5	89,707,092	80,798,459	90.07	74,516,846	92.23	6,281,613	7.77	45.01
Old 1	62,712,522	57,345,261	91.44	53,413,212	93.14	3,932,049	6.86	46.42
Old 2	46,590,704	41,788,780	89.69	38,331,156	91.73	3,457,624	8.27	40.46
Old 3	61,364,402	55,643,829	90.68	51,415,610	92.40	4,228,219	7.60	46.36
Old 4	55,361,082	49,725,122	89.82	45,615,238	91.73	4,109,884	8.27	42.44
Maximum	89,707,092	80,798,459	92.14	74,516,846	93.23	6,281,613	8.38	47.4
Mean	65,591,734	59,482,205	90.65	54,953,876	92.36	4,528,330	7.64	44.92
Minimum	46,590,704	41,788,780	89.05	38,331,156	91.62	3,457,624	6.77	40.46

The table shows the number and percentage of reads mapped to the human reference sequences. ^a^Sum of R1 and R2 reads used in the alignment. ^b^Percentage of reads used in the alignment that align to the reference genome. ^c^Percentage of reads used in the alignment that align to the reference genome in the correct relative orientation to their mate. ^d^Percentage of reads used in the alignment that align to the reference genome but whose mate does not align. ^e^Percentage of 63,152 annotated human genes with at least one read aligned.

These reads were used to estimate transcript expression of all nine samples using FPKM in order to identify the most abundant genes in tendon. Table 3 demonstrates the 25 most highly expressed genes in young and old tendon (the entire data set is in Additional file 1).

**Table 3:** **Top 25 genes**

**Gene name**	**Name**	**Log fold change**	**FDR-adjusted**	**Mean FPKM**
*RN7SK*	7SK small nuclear	0.1	1.0	22,323.9
*RN7SL2*	RNA, 7SL, cytoplasmic 2	−0.5	0.7	12,212.2
*RN7SL4P*	RNA, 7SL, cytoplasmic, pseudogene 1	−0.6	0.6	5,813.8
*RN7SL1*	RNA, 7SL, cytoplasmic 2	−1.1	0.5	3,846.0
*ANGPTL7*	Angiopoietin-like 7	0.4	0.8	3,529.0
*RNA28S5*	Ribosomal RNA 18S 6	−0.3	0.9	3,374.0
*RN7SKP255*	7SK small nuclear pseudogene 255	0.1	1.0	2,898.5
*RN7SKP203*	7SK small nuclear pseudogene 203	−0.1	1.0	2,836.7
*S100A6*	S100 calcium binding protein A6	0.1	0.9	1,950.3
*MALAT1*	Metastasis associated lung adenocarcinoma transcript 1	0.0	1.0	1,801.5
*DCN*	Decorin	0.0	1.0	1,791.5
*RN7SL5P*	RNA, 7SL, cytoplasmic, pseudogene 2	−1.3	0.4	1,774.0
*THBS4*	Thrombospondin 4	−0.8	0.6	1,368.2
*MT-CO1*	Mitochondrial cytochrome c oxidase III	−0.6	0.6	1,230.9
*TMSB10*	Thymosin beta 10	−0.3	0.7	1,220.1
*ASPN*	Asporin	−1.6	0.5	1,145.3
*MMP3*	Matrix metallopeptidase 3	2.2	0.2	1,126.7
*RNA18S5*	Ribosomal RNA 28S 5	−0.2	0.9	1,125.5
*CLU*	Clusterin	0.3	0.9	1,106.6
*MT-ND3*	Mitochondrial NADH dehydrogenase 3	−0.5	0.6	1,036.1
*LUM*	Lumican	0.0	1.0	978.9
*VIM*	Vimentin	0.2	0.9	977.1
*CILP*	Cartilage intermediate layer protein	0.3	0.9	920.8
*EEF1A1*	Eukaryotic translation elongation factor 1 alpha-like 7	0.0	1.0	891.4
*IGFBP6*	Insulin-like growth factor-binding protein 6	0.7	0.6	859.0

The table demonstrates the 25 most highly expressed genes in young and old tendon in terms of transcript expression as determined by using fragments per kilobase of exon per million fragments mapped (FPKM). FDR, false discovery rate.

### Identification of differentially expressed genes and isoforms

**Figure 1:**
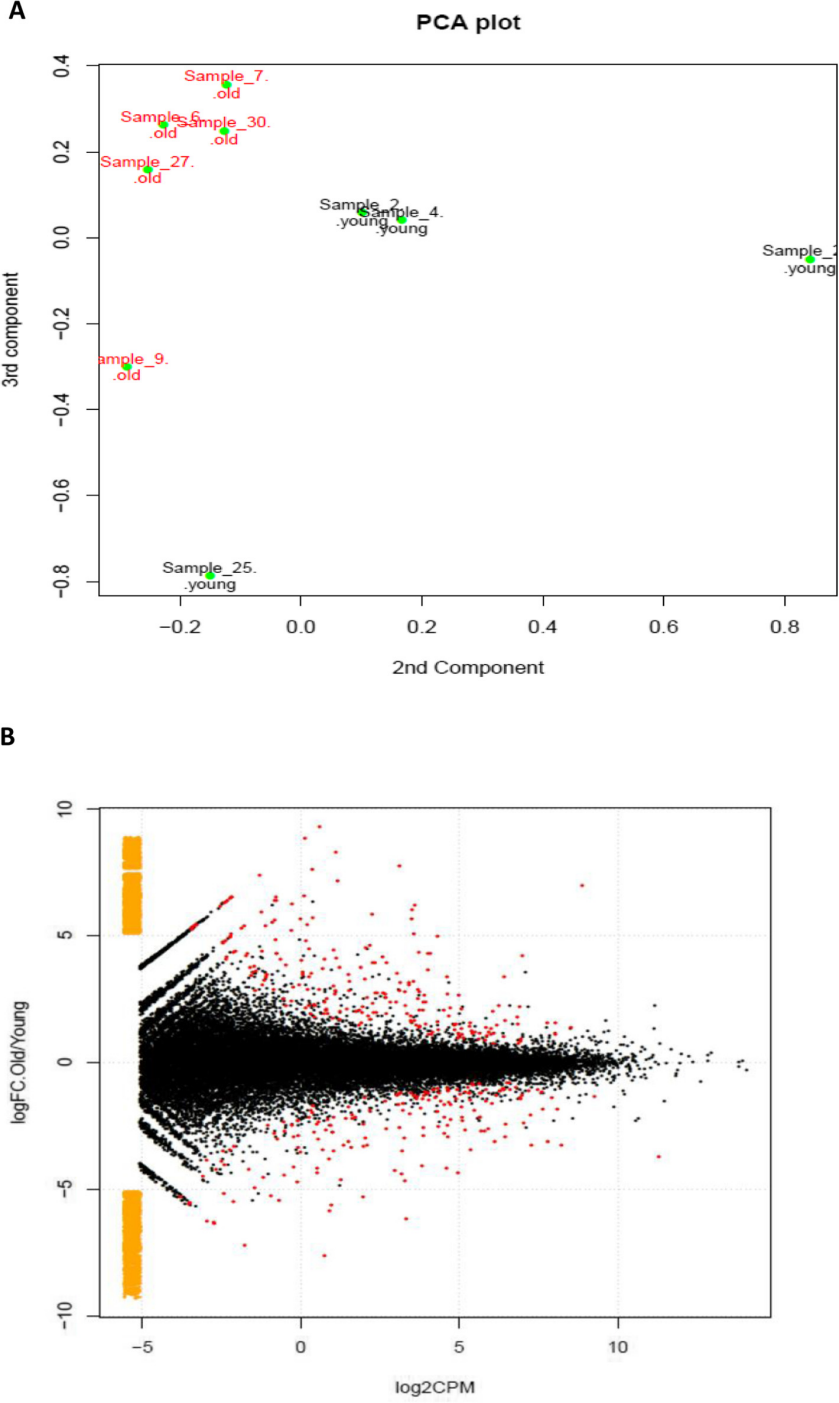
**Principal component analysis (PCA) and volcano plot of differentially abundant transcripts. (A)** PCA plot of log_2_ gene expression data showed the greatest variability in RNA-Seq data was due to the age of the donor. The second and third components are also shown as changes in the first and second components are due to differing sample sizes between young and old groups. **(B)** Volcano plot represents log_2_ FC × log_2_ CPM plot (counts per million mapped reads) CPM plot. Low expression genes (log_2_ CPM of less than −5) are coloured orange. Significant differentially expressed genes (DEGs) are coloured in red. A set of DEGs between young and old tendon was identified. With the common dispersion in edgeR [30], 325 DEGs were identified with a *P* value of less than 0.05 (red).

A principal component analysis (PCA) plot of log_2_ gene expression data indicated that the effect of age on gene expression was distinct as data were clustered in two groups (Figure 1A). Within the young group, two samples clustered together and two were independent of each other indicating more variability between young donors. Alterations in gene expression between young and old tendon demonstrated significant age-related changes. In total, the expression of 325 transcribed elements, including protein-coding transcripts and non-coding transcripts, small non-coding RNAs (snoRNAs), pseudogenes, lncRNAs, and a single microRNA, was significantly different in old compared with young tendon (±1.4 log_2_ fold change, FDR-adjusted *P* value of less than 0.05) (Figure 1B). Of these, 191 were at higher levels in the older tendon and 134 were at lower levels in the older tendon. The top 10 genes most DEG (increased and decreased) during tendon ageing are given in Table 4. The entire list of significantly DEG transcripts is presented in Additional file 2. NCBI GEO under accession number E-MTAB-2449 contains a complete list of all genes mapped. Of the 191 transcripts expressed at a higher level in old donors, 148 were known protein-coding genes. The remaining 43 genes contained 34 lncRNAs, one snoRNA, and eight pseudogenes (Table 5). Within the group where gene expression was lower in old compared with young tendon, 112 were known protein-coding genes. The remaining 22 genes contained 16 lncRNAs, one snoRNA, four pseudogenes, and a single microRNA (miRNA) (Table 6). Thus, 325 genes were input into IPA for downstream analysis, and 273 of these were mapped.

**Table 4:** **Top 10 genes with the highest and lowest log**_**2**_ **fold change when comparing young and old tendon**

**Condition increased differential expression**	**Gene symbol**	**Gene name**	**Location**	**Function**	**Log**_**2**_ **fold change**
**Old**	*CYP24A1*	Cytochrome P450, family 24, subfamily A,1	Cytoplasm	Enzyme	9.3
	*FOLH1B*	Folate hydrolase 1B	Cytoplasm	Enzyme	8.8
	*POU3F4*	POU class 3 homeobox 4	Nucleus	Transcription regulator	8.3
	*FOLH1*	Folate hydrolase 1	Plasma membrane	Peptidase	7.7
	*BRS3*	Bombesin-like receptor 3	Plasma membrane	G-protein coupled receptor	7.6
	*XIST*	X (inactive)-specific transcript (non-protein coding)	Variable	Long non-coding RNA	6.9
	*EGF*	Epidermal growth factor	Extracellular space	Growth factor	6.5
	*BRINP3*	Bone morphogenetic protein/retinoic acid inducible neural-specific 3	Secreted/mitochondria	Osteoblast differentiation factor	6.5
	*NXPH2*	Neurexophilin 2	Extracellular space	Signalling molecules	6.5
	*MYF5*	Myogenic factor 5	Nucleus	Transcription regulator	6.4
**Young**	*CYP1A1*	Cytochrome P450, family 1, subfamily A, polypeptide 1	Cytoplasm	Enzyme	−7.6
	*CLEC3A*	C-type lectin domain family 3, member A	Secreted	Cell adhesion	−6.1
	*DIRAS1*	DIRAS family, GTP-binding RAS-like 1	Plasma membrane	Enzyme	−5.9
	*SYT13*	Synaptotagmin XIII	Plasma membrane	Transporter	−5.6
	*GPR143*	G protein-coupled receptor 143	Plasma membrane	G-protein coupled receptor	−5.6
	*SLC7A14*	Solute carrier family 7 member 14	Plasma membrane	Transporter	−5.4
	*HIST 1H3A*	HISTONE 1H3A	Nucleus	Nucleosome	−5.4
	*PYCR1*	Pyrroline-5-carboxylate reductase 1	Cytoplasm	Enzyme	−5.3
	*UTS2R*	Urotensin 2 receptor	Plasma membrane	G-protein coupled receptor	−5.2
	*HBG1*	Hemoglobin, gamma A	Cytoplasm	Fe carrier	−4.9

Log_2_ fold change and q value (adjusted *P* value) were determined in edgeR. A logarithm to the base 2 of 9 is approximately a linear fold change of 3.2. Shown are the 10 genes with highest and lowest expression in old compared with young tendon samples.

**Table 5:** **Non-coding RNAs significantly overexpressed in old tendon**

**Gene accession**	**Category**	**Type of transcript**	**Log**_**2**_ **fold change**	**Description/Aliases**	**Function**
RNVU1-6	SnoRNA	Small nuclear	4.9	Variant U1 small nuclear 6	Splicesomal
RP11-578O24.2	Pseudogene	Pseudogene	6.2		Unknown
AP003041.1	Pseudogene	Pseudogene	5.3		Unknown
MKRN7P	Pseudogene	Pseudogene	4.8	Makorin ring finger protein 7	Unknown
RPS4XP22	Pseudogene	Pseudogene	4.1	Ribosomal protein S4X pseudogene 22	Unknown
RP11-346 M5.1	Pseudogene	Pseudogene	2.8		Unknown
RN7SKP234	Pseudogene	Pseudogene	2.4	RNA,7SK small nuclear pseudogene 243	Unknown
CTD-2114 J12.1	Pseudogene	Pseudogene	2.1		Unknown
AL021068.1	Pseudogene	Pseudogene	1.9		Unknown
CTD-2083E4.4	LncRNA	Novel processed	2.3		Unknown
RP3-326 L13.3	LncRNA	Novel lncRNA	7.1		Unknown
RP11-377D9.3	LncRNA	Novel lncRNA	6.3	Inc-KIAA1467-2	Unknown
RP11-71E19.2	LncRNA	Novel lncRNA	5.4	Inc-KLF15-2	Unknown
AC073636.1	LncRNA	Novel lncRNA	5.1	Inc-NFE2L2-3	Unknown
RP11-279 F6.1	LncRNA	Novel lncRNA	4.7	Inc-RPLP1-1	Unknown
AC004510.3	LncRNA	Novel lncRNA	4.1		Unknown
RP11-500B12.1	LncRNA	Novel lncRNA	4.1	Inc-TLR4-1	Unknown
AC007405.6	LncRNA	Novel lncRNA	3.8	Inc-AC007405.7.1-1	Unknown
RP11-399D6.2	LncRNA	Novel lncRNA	3.5	Inc-DMRTA1-2	Unknown
RP11-966I7.1	LncRNA	Novel lncRNA	3.5	Inc-PRKD1-8	Unknown
RP11-815 M8.1	LncRNA	Novel lncRNA	3.1	Inc-DUSP10-3	Unknown
AC003090.1	LncRNA	Novel lncRNA	2.4	Inc-NPVF5	Unknown
RP11-79H23.3	LncRNA	Novel lncRNA	2.2	Inc-FAM164A-1	Unknown
RP11-4 F5.2	LncRNA	Novel lncRNA	2.0	Inc-MCTP2-1	Unknown
RP5-1024G6.8	LncRNA	Novel lncRNA	1.9		Unknown
NKX2-1-AS1	LncRNA	Novel antisense	7.4	NKX2-1 antisense RNA 1	Unknown
RP3-326 L13.2	LncRNA	Novel antisense	6.5		Unknown
RP11-464O2.2	LncRNA	Novel antisense	5.4	Inc-LHPP-1	Unknown
AC091633.3	LncRNA	Novel antisense	5.4	Inc-MUC20-2	Unknown
RP11-711G10.1	LncRNA	Novel antisense	5.3		Unknown
SATB2-AS1	LncRNA	Novel antisense	4.2	SATB2 antisense RNA 1	Unknown
FEZF1-AS1	LncRNA	Novel antisense	4.1	FEZF1 antisense RNA 1	Unknown
SLC26A4-AS1	LncRNA	Novel antisense	3.7	SLC26A4 antisense	Unknown
RP11-160A10.2	LncRNA	Novel antisense	3.5	Inc-CLVS2	Unknown
RP4-803 J11.2	LncRNA	Novel antisense	3.4	Inc-RAB4A-1	Unknown
RP11-402 J6.1	LncRNA	Novel antisense	2.5	Inc-ALPK1-1	Unknown
RP11-710C12.1	LncRNA	Novel antisense	2.0		Unknown
XIST	LncRNA	Known lncRNA	7.0	X inactive specific transcript	X chromosomal inactivation
LINC00261	LncRNA	Known lncRNA	5.9	LINC RNA 261 Inc FOXA2-2	Cancer
TSIX	LncRNA	Known lncRNA	3.9	TSIX transcript, XIST antisense RNA	Antisense during X chromosomal inactivation
LINC00461	LncRNA	Known lncRNA	2.5	INC MEF2C-2	Unknown
DLX6-AS1	LncRNA	Known antisense	2.7	Embryonic ventral forebrain-1	Gene expression; hippocampus

Genes here have at least a ±1.4 log_2_ fold change and false discovery rate-adjusted *P* value of less than 0.05 as determined by edgeR. LncRNA, long-coding RNA; SnoRNA, small non-coding RNA.

**Table 6:** **Non-coding RNAs with significantly reduced expression in old tendon**

**Gene accession**	**Category**	**Type of transcript**	**Log**_**2**_ **fold change**	**Description/Aliases**	**Function**
LINC00957	LncRNA	Novel lncRNA	−1.73	Long intergenic non-coding RNA 957	Unknown
ZNF667-AS1	LncRNA	Novel lncRNA	−1.82	ZNF667 antisense RNA 1 (head to head)	Unknown
RP11-308 N19.1	LncRNA	Novel lncRNA	−2.43		Unknown
AF131215.9	LncRNA	Novel sense intronic	−2.78		Unknown
CTB-113P19.1	LncRNA	Novel antisense	−2.93	Inc-G3BP1-2	Unknown
AF131215.2	LncRNA	Novel sense intronic	−3.24		Unknown
MAFG-AS1	LncRNA	Known antisense	−3.45	MAFG antisense RNA 1 (head to head)	Unknown
AC020571.3	LncRNA	Novel antisense	−3.50	Inc-CCDC150-1	Unknown
AC012613.2	LncRNA	Novel antisense	−3.86	Inc-IL17B-3	Unknown
RP11-923I11.1	LncRNA	Novel antisense	−3.91	Inc-SCN8A-1	Unknown
RP11-270 M14.5	LncRNA	Novel lncRNA	−4.01	Inc-TTLL5-1	Unknown
CTD-2540B15.9	LncRNA	Novel lncRNA	−4.50	Novel lincRNA	Unknown
CTD-3049 M7.1	LncRNA	Novel lncRNA	−4.53	Inc-RGMA7	Unknown
RP5-1198O20.4	LncRNA	Novel lncRNA	−5.25	Inc-KLF17-1	Unknown
RP11-300E4.2	LncRNA	Novel antisense	−6.24	Inc-JPH1_5	Unknown
RP11-445 L6.3	LncRNA	Novel lncRNA	−6.31	Inc-TNC-2	Unknown
MIR1245A	MiRNA	Known miRNA	−2.15		Unknown
MXRA5P1	Pseudogene	Pseudogene	−3.52	MXRA5P1	Unknown
RNY3P2	Pseudogene	Pseudogene	−5.30	RNA Ro-associated Y3 pseudogene 2	Unknown
RP11-494 K3.2	Pseudogene	Pseudogene	−6.36		Unknown
CTC-260E6.10	Pseudogene	Pseudogene	−7.23		Unknown
Y_RNA	SnoRNA	Novel misc RNA	−5.53		DNA replication and cell proliferation

Terms are derived from Ensemble [46] and Vega [47]. ‘Antisense’ overlaps the genomic span of a protein-coding locus on the opposite strand. ‘Known’ indicates identical to known cDNA or proteins from the same species and has an entry in a model database. ‘Novel’ indicates identical or homologous to cDNAs from the same species or proteins from all species. ‘Processed transcript’ does not contain open reading frame and cannot be placed in any other category. ‘Pseudogene’ indicates homology to protein but from a disrupted coding sequence and an active homologous gene can be found at another locus. ‘Sense intronic’ has a long non-coding transcript in introns of a coding gene that does not overlap any exons. LncRNA, long non-coding RNA (which can be further classified as LINCRNA, which is a long interergenic non-coding RNA locus of more than 200 base pairs); miRNA, microRNA; SnoRNA, small non-coding RNA.

**Figure 2:**
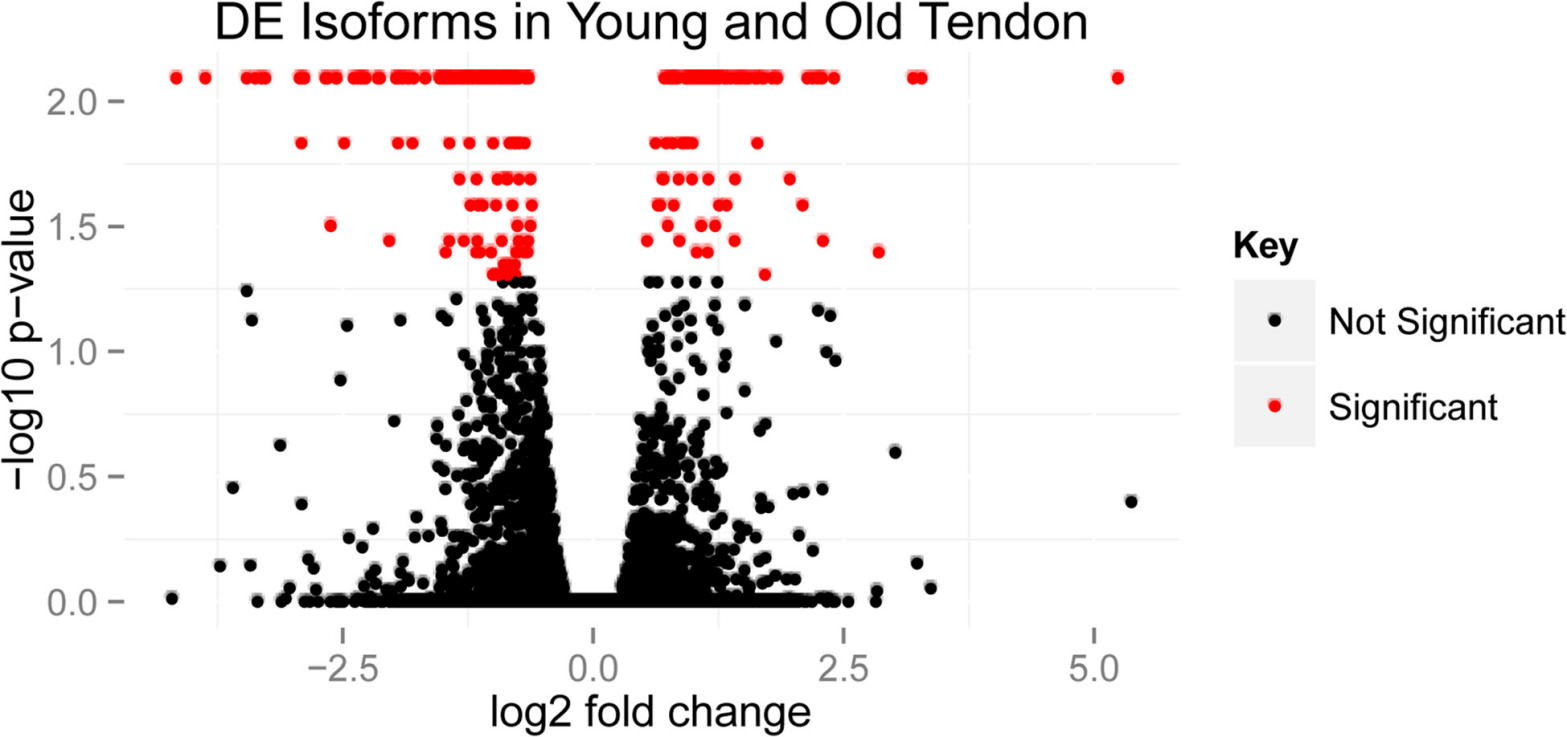
**Volcano plot showing significant differentially expressed (DE) isoforms between young and old tendon.** Following assembly with Cufflinks of alternative splicing transcripts [36], significant DE transcripts were identified with q values (*P* value adjusted to false discovery rate) of less than 0.05. Red spots represent significant DE isoforms, and black spots are for non-DE isoforms.

The analysis identified a number of transcript isoforms expressed in tendon, some of which were differentially expressed between young and old groups of tendon (Figure 2). In total, 183,660 isoforms were detected in young and 191,673 isoforms were detected in old tendon. Among these, 21,193 isoforms were detected only in young and 29,206 isoforms only in old. Sixty-three known isoforms were upregulated in old tendon, with 80 downregulated with an FDR-adjusted *P* value of less than 0.05 and ±1.4 log_2_ fold regulation. The top 10 up- and down-regulated isoforms are presented in Table 7. The entire list of significantly DEG isoforms is presented in Additional file 3.

**Table 7:** **List of top 10 up-/down-regulated isoforms in ageing tendon**

**Comparison**	**Gene**	**Locus**	**Log**_**2**_ **(fold change)**	**q value**
Higher in old	PM20D2	6:89855768-89875284	0.54	0.04
	ILF3-AS1	19:10762537-10764520	0.62	0.01
	PERP	6:138411922-138428648	0.65	0.03
	ODC1	2:10580093-10588630	0.67	0.03
	PRKAA2	1:57110994-57181008	0.68	0.03
	PNRC1	6:89790469-89794879	0.69	0.02
	SYT11	1:155829299-155854990	0.70	0.02
	F13A1	6:6144317-6321246	0.71	0.01
	CITED2	6:139693392-139695757	0.72	0.01
	LDB1	10:103867316-103880210	0.73	0.01
Lower in old	HLA-DRB5	6:32485119-32498064	−4.72	0.01
	MYH1	17:10286448-10527201	−4.52	0.01
	CRABP2	1:156669397-156675608	−4.44	0.03
	CPXM1	20:2774714-2781283	−4.29	0.01
	COL3A1	2:189839045-189877472	−4.16	0.01
	COL3A1	2:189839045-189877472	−3.87	0.01
	ADAM12	10:127700949-128077024	−3.84	0.01
	CAPN6	X:110488330-110513751	−3.72	0.03
	COL1A1	17:48260649-48278993	−3.46	0.01
	FAP	2:163018279-163101661	−3.37	0.01

The differentially expressed genes (DEGs) in young versus old tendon were determined by Cuffdiff. The fold change is the ratio of fragments per kilobase of exon per million fragments mapped (FPKM) of those genes in young to FPKM of those genes in old tendon. The significant DEGs (q values of less than 0.05) calculated with a Benjamini-Hochberg correction were ranked on their log_2_ fold change, and 10 genes with the highest or lowest fold changes are shown in the table.

### Age-related changes in transcription factors

There was an increase in the expression of 12 transcription factors in tendon derived from older donors compared with young: ALX homeobox (ALX1), insulin gene enhancer protein 1 (ISL1), lim homeobox 9 (LHX9), myocardin (MYOCD), POU domain, class 3, transcription factor 4 (POU3F4), POU3F3, paired box transcription factor 3 (PAX3), PAX6, PAX9, thyroid transcription factor 1 (NKX2-1), NKX6-1, and dachshund family transcription factor 2 (DACH2). In comparison, there was an increase in only three in younger donors: basic helix-loop-helix family, member e40 (BHLHE40), early growth response 2 (EGR2), and zinc finger of the cerebellum 3 (ZIC3).

### Differentially expressed genes and network analysis

DEGs (325) and differentially expressed transcript isoforms (143) associated with ageing were analysed together in IPA with the following criteria: *P* value of less than 0.05 and 1.4 log_2_ fold change. Network-eligible molecules were overlaid onto molecular networks based on information from the ingenuity pathway knowledge database and networks generated based on connectivity. (See Additional file 4 for all identified networks and their respective molecules.)

**Figure 3:**
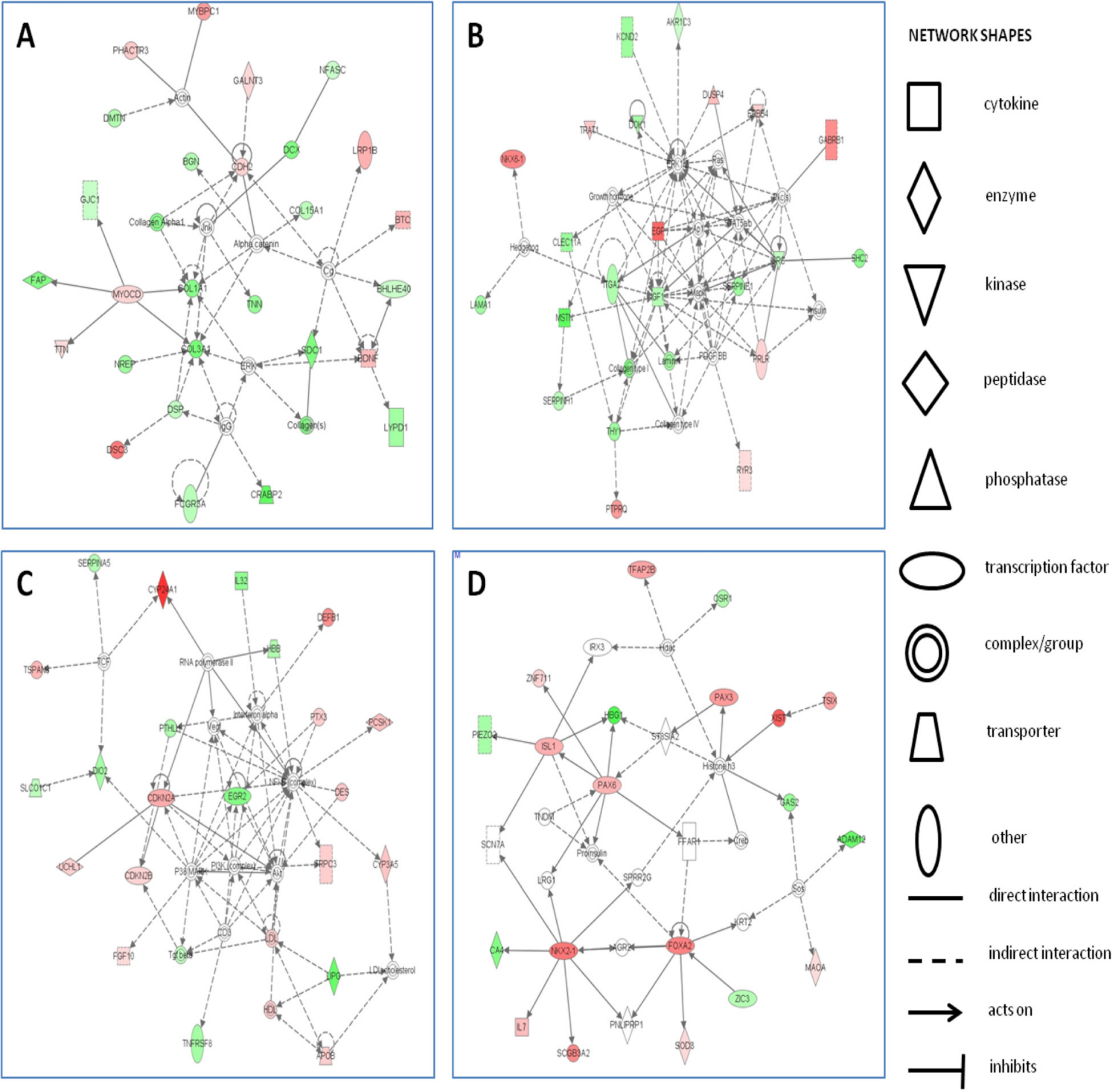
**Top-scoring networks derived from the 325 genes differentially expressed in ageing. (A)** Ingenuity pathway analysis (IPA) identified from cellular function and maintenance as the principal associated network functions with scores of 43. **(B)** The second top-scoring network was a cellular growth and proliferation, with scores of 32. **(C)** IPA identified cell cycle and skeletal and muscular system development function with a score of 32. **(D)** The fourth top-scoring network was cellular development, with a score of 28. Figures are graphical representations between molecules identified in our data in their respective networks. Green nodes indicate upregulated gene expression in older tendon; red nodes indicate downregulated gene expression in older tendon. Intensity of colour is related to higher fold change. The key to the main features in the networks is shown.

The top four scoring networks for genes differentially expressed with tendon age were from cellular function and maintenance, cellular growth and proliferation, cellular cycling, and cellular development (Figure 3). Significant IPA canonical pathways are demonstrated in Table 8, and the associated molecules of the top canonical pathways identified are in Additional file 5. These include hepatic fibrosis, oestrogen biosynthesis, and transcriptional regulatory networks in embryonic stem cells. Interestingly, skeletal and muscular disorders were identified as one of the top diseases associated with the gene set (Additional file 6).

**Table 8:** **A number of ingenuity pathway analysis canonical pathways were significantly affected in ageing tendon**

**Name of canonical pathway**	***P*** **value**	**Ratio**
Hepatic fibrosis/Hepatic stellate cell activation	5.69 × 10^−4^	8/142 (0.056)
Oestrogen biosynthesis	1.61 × 10^−3^	4/38 (0.105)
Transcriptional regulatory network in embryonic stem cells	1.95 × 10^−3^	4/40 (0.1)
Glioblastoma signalling	3.66 × 10^−3^	7/159 (0.044)
Bupropion degradation	4.89 × 10^−3^	3/26 (0.115)

The significance of the association between the data set and the canonical pathway was measured by using a ratio of the number of molecules from the data set that mapped to the pathway divided by the total number of molecules that map to the canonical pathway. Fisher’s exact test was used to calculate *P* values.

### Functional annotation of up- and down-regulated isoforms

There was a reduction in the DEG isoforms of 32 genes (representing 15% of the data set) relating to the ECM, degradative proteases, cytokines, and growth factors in tendon derived from older donors compared with young donors. In comparison, there was an increase in only two ECM genes (representing 1.3% of the data set) in older donors (data not shown). DAVID identified significant gene ontology (GO) terms in the upregulated and downregulated set of transcript isoforms (Table 9) with only two terms ‘secreted’ and ‘signal’ overlapping between the two groups. Interestingly, other terms are strikingly different between the upregulated and downregulated isoform data sets. Several of the top GO terms identified in downregulated isoforms in old tendon relate to collagen and post-translational modification of collagen (for example, hydroxylation, hydroxylysine, hydroxyproline, and triple helix).

**Table 9:** **Significant gene ontology terms annotated according to DAVID’s SP-PIR-Keywords**

**Term**	**Count**	**Percentage**	***P*** **value**
GO terms identified in downregulated isoforms in old tendon			
Signal	88	45.1	6.30 × 10^−18^
Secreted	58	29.7	7.04 × 10^−15^
Extracellular matrix	24	12.3	3.25 × 10^−14^
Glycoprotein	85	43.6	2.72 × 10^−9^
Collagen	12	6.2	1.62 × 10^−7^
Trimer	7	3.6	8.71 × 10^−6^
Hydroxylation	9	4.6	2.22 × 10^−5^
Triple helix	7	3.6	2.00 × 10^−5^
Hydroxylysine	7	3.6	2.00 × 10^−5^
Disulfide bond	57	29.2	1.85 × 10^−5^
Cell adhesion	18	9.2	3.57 × 10^−5^
Hydroxyproline	7	3.6	4.38 × 10^−5^
Ehlers-Danlos syndrome	5	2.6	7.34 × 10^−5^
Angiogenesis	6	3.1	0.011506
Metalloprotease	8	4.1	0.016954
Pyroglutamic acid	5	2.6	0.021871
GO terms identified in upregulated isoforms in old tendon			
Secreted	30	24.2	1.48 × 10^−4^
Plasma	7	5.6	0.003767
Signal	39	31.5	0.006761
Acute phase	4	3.2	0.052997

Gene ontology (GO) terms were revealed in upregulated and downregulated differentially expressed gene isoforms of tendon ageing. *P* value represents Benjamini-Hochberg corrected *P* value. DAVID, Database for Annotation, Visualization and Integrated Discovery.

### Confirmation of DEG by using qRT-PCR measurements of selected genes

To validate the RNA-Seq technology, selected gene expression differences noted in the RNA-Seq analysis were re-measured by using reverse transcription and qRT-PCR. This was performed on the original RNA from all donors used to perform the RNA-Seq experiment (Table 10) and an independent cohort (Additional file 7A). All genes were found to have comparable results with RNA-Seq data; for instance, genes identified as having an increase in expression in older samples in the RNA-Seq experiment also gave increased expression relative to RPS16 following qRT-PCR. Statistical significance was tested by using Student’s *t* test. Two genes whose expressions were not significantly altered in RNA-Seq results—aggrecan (ACAN) and MMP3—were also unaltered when assessed with qRT-PCR. Gene expression analysis using qRT-PCR of an independent cohort found similar results. Validation of differential isoform expression by using qRT-PCR was in general concordance with RNA-Seq (Additional file 7B). In all cases, the level of expression varied between the two platforms.

**Table 10:** **Real-time polymerase chain reaction analysis of 14 selected genes reveals good correlation with RNA-Seq results**

**Gene name**	**RNA-Seq results**			**RT-PCR results**		
				**Age**		***P*** **value**
	**Differential expression**	**Significant log**_**2**_ **fold change**	**q value**	**Young**	**Old**	
*EGF*	Higher in old	6.50	2.06 × 10^−8^	0.003 ± 0.002	0.01 ± 0.007	0.04
*POU3F4*		8.20	7.79 × 10^−16^	0.021 ± 0.015	0.245 ± 0.147	0.02
*MYF5*		6.30	0.003	0.061 ± 0.057	0.25 ± 0.07	0.01
*TGFB3*	Lower in old	−1.60	0.035	10.717 ± 6.022	0.897 ± 0.34	0.05
*MMP16*		−2.30	0.022	0.107 ± 0.087	0.014 ± 0.02	0.07
*COL3A1*		−3.70	0.008	13.696 ± 17.535	1.05 ± 0.594	0.27
*MYH1*		−4.60	0.003	0.288 ± 0.155	0.14 ± 0.101	0.23
*IGF1*		−2.10	0.004	2.094 ± 1.234	0.483 ± 0.278	0.04
*COL1A1*		−3.30	0.009	47.968 ± 32.676	8.515 ± 9.154	0.42
*MMP3*	No change	Not significant	1.000	51.181 ± 362.11	99.323 ± 66.10	0.15
*ACAN*			1.000	1.439 ± 1.43	0.848 ± 0.99	0.66
*XIST*	Higher in old	6.90	0.000	0.827 ± 0.712	7.41 ± 3.465	0.03
*LINC00957*	Lower in old	−1.70	0.036	1.044 ± 0.584	0.363 ± 0.174	0.08
*RP11.308 N19.1*		−2.430	0.040	0.02 ± 0.065	0.005 ± 0.001	0.03

Values for real-time polymerase chain reaction (RT-PCR) are the mean ± standard deviation of relative expression levels normalised to expression of RSP16 (to two decimal places). Statistical significance was tested by using Student’s *t* test. RT-PCR results are expressed as 2^-DCT. ACAN, aggrecan; COL1A1, collagen type 1 alpha 1; COL3A1, collahen type 3 alpha 1; EGF, epidermal growth factor; IGF1, insulin growth factor 1; LINC00957 long intergenic non-protein coding RNA 957; MMP3, matrix metalloproteinase; MMP16, matrix metalloproteinase 16; MYF5, myogenic factor 5; MYH1, myosin heavy chain 1; POU3F4, POU class 3 homeobox 4; RP11.308 N19.1, Inc-ZNF462-2; TGFB3, transforming growth factor β.

## Discussion

Ageing is recognised as a significant risk factor for tendon injury; however, knowledge of changes to the transcriptome of tendon cells has previously been limited to that gained from quantitative PCR [5,48,49] and microarray studies on tendinopathic human [50,51] and rat tissue [17,52]. In this study, we report for the first time the use of the RNA-Seq technique to undertake deep transcriptome profiling of young and old macroscopically normal human Achilles tendon. Importantly, validation studies using qRT-PCR demonstrated high correlation between methodologies and demonstrated reproducibility using a different donor set. One of the many advantages of RNA-Seq over microarrays is that it enables *de novo* analysis of transcripts, including novel transcripts. In this study, we were able to identify and quantify protein-coding transcripts, alternatively spliced isoforms, lncRNAs, pseudogenes, and small regulatory RNAs, including small nucleolar RNAs (snoRNA) and an miRNA. The age of the donor accounted for most of the variability in the data, although PCA identified more variability between young donors. We did not have access to detailed medical history and lifestyle factors for the patients in this study, so we are unable to determine whether other factors explain the variability more precisely.

Tendon is characterised by a large amount of ECM interspersed around a relatively sparse population of cells. The main component of the matrix is the fibril-forming type I collagen, which composes about 70% of the dry weight of the matrix [53]. Minor collagen types include other fibril-forming collagens, type III and V; fibril-associated collagens, type XII and XIV; and type VI collagen. As expected, these were the main collagen genes we identified in the transcriptome of the Achilles tendon tissue, albeit at relatively low levels. The non-collagenous component of tendon is rich in small leucine-rich proteoglycans (SLRPs), including decorin, biglycan, fibromodulin, and lumican, and the glycoproteins COMP, lubricin, tenomodulin, and tenascin C [54]. Interestingly, the results of this study show that decorin was by far the most highly expressed ECM gene across the samples in comparison with relatively low levels of collagen transcripts. Lumican was the next most highly expressed ECM protein followed by fibromodulin and COMP. These results are in line with our recent proteomics study in which decorin was the second most abundant ECM protein in a guanidine soluble extract of equine flexor tendon [55]. Degradation of the ECM is accomplished by a family of MMPs along with other proteases, and we identified expression of collagenases, stromelysins, gelatinases, and aggrecanases, although in general the levels of expression were low. An exception to this was MMP3, a stromelysin responsible for proteoglycan degradation, which was one of the most abundant transcripts, again supporting the finding of a higher turnover of non-collagenous proteins.

Ageing results in changes to the tendon ECM composition, although these are poorly defined at present and the impact on tendon mechanical properties is not clear as some studies report increased stiffness with ageing [56,57] whereas others report a decrease [58,59]. A recent study using equine flexor tendon found that, although the mechanical properties of the gross structure and the component fascicles did not change with age, the inter-fascicular matrix became stiffer. Given this finding, we expected to find differential expression of ECM transcripts, particularly those enriched in the inter-fascicular matrix. The differential gene expression analysis showed no regulation of proteins likely to be enriched in the inter-fascicular matrix or inter-fibrillar proteins [54]. The alpha 1 chain of type I collagen and alpha 1 chain of type III collagen were identified as having reduced expression in the old age group, although this lost statistical significance when measured by qRT-PCR on a larger sample set. For the most part, these data do not support our original hypothesis that tendon ageing results in reduced expression of genes relating to ECM, degradative proteases, cytokines, and growth factors, unlike changes evident in ageing cartilage [24].

Tendon disease, which has a clear association with ageing, has been the focus of several gene expression studies. Generally, findings in these studies are in keeping with the hypothesis of increased matrix turnover, with an imbalance favoring catabolism. For example, various studies have demonstrated increased expression of collagen 1 alpha 1 (COL1A1) [5,48,49] and proteins more typical of cartilage COL2A1, aggrecan, and SOX9 [5,52]. Tendinopathic samples show an upregulation of various MMPs, including MMP23 [5,51], a disintegrin and metalloproteinase 12 (ADAM12) [5,50,51], and downregulation of MMP3. The results of our study are in stark contrast to this with very low expression levels for COL2A1, aggrecan, SOX9, most MMPs (except MMP3), and a significant downregulation of ADAM12 in the old group. Therefore, the results suggest that degeneration is not an inevitable consequence of ageing and that ageing and disease-associated degeneration are distinct processes.

In this study, we identified DEG gene sets with ageing related to a dysregulation of cellular function and maintenance, cellular growth and proliferation, cellular cycling, and cellular development. Therefore, these changes suggest that the cellular component of tendon may lose the ability to respond appropriately to mechanical and chemical signals. Other studies have linked cellular senescence, a state of irreversible growth arrest, in a small subset of cells (progenitor cells) in tendon with tendon ageing [60]. A senescence phenotype has been described, although no marker of senescence identified thus far is entirely specific to the senescent state [9]. Most senescent cells express p16(INK)4a, which is not commonly expressed by quiescent or terminally differentiated cells [9]. In this study, p16(INK)4a was expressed at higher levels in the old group, although transcript levels overall were low, which may indicate that a small subpopulation of cells is responsible for the difference. Senescent cells have been shown to contribute to an inflammatory profile, and the term ‘inflamm-aging’ has been coined [13]. Studies have shown upregulation of inflammatory mediators such as cytochrome oxidase 2 (COX2), interleukin 6 (IL-6), and prostaglandin E2 (PGE2) and downregulation of the lipoxin A4 (LXA4) receptor FPR2 (formyl peptide receptor 2)/ALX in human or equine tendinopathic tissue [14,49]. Inflammatory pathways, however, were not recognised in our GO mapping of DEGs between young and old groups in this study.

An interesting finding in this study was the differential expression of isoforms and those with reduced expression in the older tendons mapping to ECM, degradative proteases, cytokines, and growth factors. AS is a significant regulatory mechanism in gene expression as it enables versatility at the post-transcriptional level accounting for proteome complexity and may affect up to 92% of human genes [61]. Differences between isoforms of the same protein extend from a complete loss of function, acquiring a new function to subtle modulations, the latter observed in the majority of cases [62]. Few AS events have been reported in tendon to date. Those that have include versican, in which AS may contribute to changes in ECM structure and function in tendinopathies [48]; lubricin, which is location-dependent [63]; and insulin-like growth factor 1, which is mechanical stress-dependent [64]. The isoforms showing the greatest difference between young and old tendon groups in our study (for example, COL1A1, COL3A1, and ADAM12) are recognised as some of the most important proteins for tendon function and the relevance of these isoforms requires further investigation.

The results of our study have yielded new information relating to tendon cell phenotype and to the ageing process, identifying transcripts that are not generally recognised as being important in tendon. For example, the gene most highly expressed, disregarding ribosomal proteins, was angiopoetin-like 7 (ANGPTL7). This protein has previously been identified as highly expressed in microarray analysis of human tendinopathic tissue from various tendons [50]. Angiopoetins are involved in angiogenesis [65], inflammation, and glucose [66] and lipid [67] metabolism. In the cornea, ANGPTL7 may function as negative regulator of angiogenesis, contributing to the avascular properties of the tissue [68], whereas in the human ocular trabecular meshwork cells, it has a role in the organization of the ECM [69]. Thus, we suggest that ANGPTL7 may have a role in maintaining the relatively avascular nature of tendon tissue and in the organisation of the ECM. This represents an area for further investigation.

One of the limitations of this study is that the samples were taken from patients with malignant disease. We consider that it is very unlikely that this has influenced the results as samples are taken only when the tumour is at a site distant to the tendon and the tendon is macroscopically normal; however, we cannot rule out the possibility that some of the genes showing high expression, such as metastasis associated lung adenocarcinoma transcript 1 (MALAT1), ANGPITL7, and S100A6, are related to the disease state.

Another point of interest was the expression of genes associated with muscle cells. For example, we observed a reduced DEG of myosin heavy chain 1 and an increase DEG of myogenic factor 5 (MYF5) and MYOCD. Our previous studies in ageing cartilage also identified DEG of muscle-related genes: myosin heavy chain 2, myosin 3A, and myosin 1B, which were all reduced in cartilage ageing [24]. Samples of Achilles tendon were taken at a region far removed from muscle insertion, and the identification of muscle genes in cartilage and tendon is unlikely to be due to inadvertent inclusion of muscle tissue.

Interestingly, there was an increase in the expression of a large set of transcription factors in old compared with young tendon. In *Caenorhabditis elegans* [70] and a number of tissues, including heart [71] and brain [72], transcription factors have been implicated in ageing. Interestingly, deacetylates Nk2 homeobox 1 (NKX2-1), a transcription factor showing upregulation in old tendon in our study, is involved in neuronal activation in dorsomedial and lateral hypothalamic nuclei, a function thought to contribute to a more ‘youthful’ physiology during ageing [73]. Conversely, an isoform of scleraxis (SCX), a critical transcription factor in tendon development [74], was reduced in old tendon. In addition, the reduced expression in old tendon of EGR2 and AS EGR1, both of which are required for tendon differentiation [75], may affect tendon repair [76].

We identified eight pseudogenes showing upregulation in the old group of tendons. Pseudogenes have similar sequences to their counterpart coding genes, but owing to mutation/deletion or insertion of nucleotides they cannot be transcribed. It is hypothesised that pseudogenes act as post-transcriptional regulators of the corresponding parental gene [74]. In other studies, pseudogenes have been identified as increasing with age, such as pseudogene cyclin D2 in the ovary [76], and recent work has indicated that they may have a role in inflammation [77]. This provides an exciting new frontier to explore in ageing research, and further work is required to determine whether any of the pseudogenes identified in this study have functional significance.

Our study is the first to profile lncRNAs in tendon. LncRNAs are a large and functionally heterogeneous class of RNAs with a length of more than 200 nucleotides. They have been shown to regulate mRNA transcription, splicing, stability, translation, and epigenetic modification, providing a complex spectrum of gene regulatory functions [78], and a number of studies have identified roles for lncRNAs in ageing [22,79]. In this study, lncRNAs were shown to be DEGs in ageing tendon, and 34 showed upregulation in old tendon. In musculoskeletal disease, relatively little work interrogates the role lncRNAs in tissue physiology and disease except for a few studies in cartilage/OA [80-82] and muscle (reviewed [83]) and an osteosarcoma study [84]. The lncRNA transcriptome signatures in ageing tendon provide an interesting set of genes for further studies to determine their role in tendon ageing and disease.

## Conclusions

Our study is the first to interrogate tendon by using RNA-Seq. We demonstrate dynamic alterations in RNA with age, at numerous genomic levels, which indicate changes in the regulation of transcriptional networks. Further extensive follow-up analysis of modulator genes, splice variants, and non-coding RNAs found in this study may be useful in understanding tendon ageing.

## Additional files

Additional file 1:
**Transcript expression in all samples.** A complete list of significantly expressed genes and DAVID (Database for Annotation, Visualization and Integrated Discovery) analysis of the expression patterns is shown in a complete table of all transcript expression as an estimate of overall abundance of genes in ageing tendon. Reads were used to estimate transcript expression of all nine samples by using fragments per kilobase of exon per million fragments mapped (FPKM) in order to identify the most abundant genes in tendon. Based on the gene annotation file, a gene contains certain features. The total feature length of a gene is known (GFL). The count numbers obtained from read alignment for a gene depend on the real expression level of the gene and its GLF value. Therefore, counts cannot be used for comparing the expression level among genes. The sequence depth also influences the count values for given gene expression level. FPKM corrects for these two effects. FC, fold change; FDR, false discovery rate; PV, P-value; GFL, Gene feature length SD, standard deviation. Additional file 2:
**The entire set of significantly differentially expressed gene transcripts.** Significant differentially expressed genes have a log_2_ fold change (FC) ± 1.4 and false discovery rate (FDR)-adjusted *P* <0.05. Highest in old (gene names marked in red) are genes more highly expressed in old tendon compared with young tendon, and lowest in old (marked in blue) are genes less expressed in old tendon compared with young tendon. Additional file 3:
**The entire list of significantly differentially expressed gene isoforms.** A complete table of significant differentially expressed isoforms with a log_2_ fold change ± 1.4 and false discovery rate-adjusted *P* <0.05 is shown. FPKM, fragments per kilobase of exon per million fragments mapped. Additional file 4:
**All identified networks and their respective molecules.** Network-eligible molecules were overlaid onto molecular networks. All identified networks and their respective molecules are tabulated. Additional file 5:
**Associated molecules of the top canonical pathways.** Molecules in the tendon RNA-Seq data set associated with the top five canonical pathways are shown. Additional file 6:
**Skeletal and muscular disorders were the top diseases associated with the gene set.** Disease annotation and associated *P* value, activation z-score, and molecules associated with skeletal and muscular disorders are shown. Additional file 7:
**Quantitative real-time polymerase chain reaction (qRT-PCR) analysis of 12 selected genes reveals good correlation with RNA-Seq results in an independent cohort.** Values for qRT-PCR are the mean ± standard deviation of relative expression levels normalised to expression of RPS16. Statistical significance was tested by using Student’s *t* test. ACAN, aggrecan; COL1A1, collagen type I alpha 1; COL3A1, collagen type III, alpha 1; EGF, epidermal growth factor; FC, fold change; IGF1, insulin growth factor 1; LINC00957, long intergenic non-protein coding RNA 957; MMP-3, matrix metalloproteinase 3; MMP-16, matrix metalloproteinase 16; MYF5, myogenic factor 5; MYH1, myosin heavy chain 1; POU3F4, POU class 3 homeobox 4; TGFβ3, transforming growth factor β3; XIST, X inactive specific transcript.

## Abbreviations

ADAM12: a disintegrin and metallopeptidase domain 12
ALX: ALX homeobox
ANGPTL7: angiopoetin-like 7
AS: alternative splicing
bp: base pair
COL: collagen
DAVID: Database for Annotation, Visualization and Integrated Discovery
DEG: differentially expressed gene
ECM: extracellular matrix
EGR2: early growth response protein
FDR: false discovery rate
FPKM: fragments per kilobase of exon per million fragments mapped
GO: gene ontology
IL: interleukin
IPA: Ingenuity Pathway Analysis
lncRNA: long non-coding RNA
miRNA: microRNA
MMP: matrix metallo-proteinase
MYOCD: myocardin
NCBI GEO: National Center for Biotechnology Information Gene Expression Omnibus
NKX2: thyroid transcription factor 1
PAX: paired box transcription factor
PCA: principal component analysis
PCR: polymerase chain reaction
qRT-PCR: quantitative real-time polymerase chain reaction
rRNA: ribosomal RNA
RSP: ribosomal protein
snoRNA: small non-coding RNA.
