# Supplementary material for: Transcriptome analysis of ageing in uninjured human Achilles tendon
Source: Arthritis Res Ther. 2015 Feb 18;17(1):33. doi: 10.1186/s13075-015-0544-2 (PMC4355574; doi:10.1186/s13075-015-0544-2)
Supplement: Additional file 7: — Quantitative real-time polymerase chain reaction (qRT-PCR) analysis of 12 selected genes reveals good correlation with RNA-Seq results in an independent cohort. Values for qRT-PCR are the mean ± standard deviation of relative expression levels normalised to expression of RPS16. Statistical significance was tested by using Student’s t test. ACAN, aggrecan; COL1A1, collagen type I alpha 1; COL3A1, collagen type III, alpha 1; EGF, epidermal growth factor; FC, fold change; IGF1, insulin growth factor 1; LINC00957, long intergenic non-protein coding RNA 957; MMP-3, matrix metalloproteinase 3; MMP-16, matrix metalloproteinase 16; MYF5, myogenic factor 5; MYH1, myosin heavy chain 1; POU3F4, POU class 3 homeobox 4; TGFβ3, transforming growth factor β3; XIST, X inactive specific transcript. [file 13075_2015_544_MOESM7_ESM.docx]

**Supplementary File 7 RT-qPCR analysis of 12 selected genes reveals good correlation with RNA-Seq results in an independent cohort.**  Values for RT-qPCR are the mean ± SD of relative expression levels normalised to expression of RPS16. Statistical significance was tested using Student’s T test EGF= epidermal growth factor; POU3F4= POU class 3 homeobox 4; MYF5= myogenic factor 5; TGFβ3= transforming growth factor β3; MMP-16= matrix metalloproteinase 16; COL3A1= collagen type III, alpha 1; MYH1= myosin heavy chain 1; IGF1 = insulin growth factor 1; COL1A1= collagen type I alpha 1; MMP-3 = matrix metalloproteinase 3;, ACAN = aggrecan; XIST = X inactive specific transcript; LINC00957 = long intergenic non-protein coding RNA 957.

| Gene name | RNA-Seq Results | | | RT-PCR Results | | |
| --- | --- | --- | --- | --- | --- | --- |
|  |  |  |  | Age | | p-value |
|  | Differential expression | Significant Log_2_FC | q-value | Young | Old |  |
| EGF | higher in old | 6.50 | 2.06E-08 | 0.001563±0.0017 | 0.0653±0.06 | 0.07 |
| POU3F4 |  | 8.20 | 7.79E-16 | 0.02387±0.0018 | 0.2999±0.237 | 0.01 |
| MYF5 |  | 6.30 | 0.003 | 0.38657±0.269 | 0.2999±0.238 | 0.07 |
| TGFB3 | lower in old | -1.60 | 0.035 | 3.756±2.29 | 0.773±0.88 | 0.10 |
| MMP16 |  | -2.30 | 0.022 | 0.139±0.056 | 0.045±0.034 | 0.07 |
| COL3A1 |  | -3.70 | 0.008 | 0.797±0.89 | 0.099±0.123 | 0.25 |
| MYH1 |  | -4.60 | 0.003 | 0.299±0.25 | 0.602±0.55 | 0.18 |
| IGF1 |  | -2.10 | 0.004 | 0.571±0.75 | 0.023±0.04 | 0.28 |
| COL1A1 |  | -3.30 | 0.009 | 1570.6±2712.25 | 32.68±27.9 | 0.05 |
| MMP3 | no change | not significant | 1.000 | 13.5±17.23 | 99.32±66.1 | 0.15 |
| ACAN |  |  | 1.000 | 0.54±0.764 | 1.162±1.67 | 0.54 |
| XIST | higher in old | 6.90 | 0.000 | 0.46±0.66 | 2.847±4.57 | 0.36 |
| LINC00957 | lower in old | -1.70 | 0.036 | 0.882±0.738 | 1.923±2.16 | 0.39 |
